# A Synopsis of a Case of Hæmorrhoids

**Published:** 1890-02

**Authors:** 


### A SYNOPSIS OF A CASE OF HÆMORRHOIDS.

DEAR EDITOR: In your January number a case of hæmorrhoids is recorded treated by a homœopathic *physician*. Without going into the miserable innuendoes leveled apparently against a fellow-physician of the same school, who for some reason he has a quarrel with, let us look at the kind of treatment this wonderful expounder of the homœopathic law adopts.

The first prescription is given October 16th, evidently on the *one* symptom that the patient is "worse when sitting or lying." Now, I think we can hardly venture to call such a symptom a "key-note," although Phosphorus is the only medicine put against that symptom in the Repertory. Take any case of *painful* piles, it is almost impossible to sit down without pain, and lying down needs just as much care. Again Phosphorus *I believe does not give shooting pains in the vagina near* orifice. Now Sulphur gives shooting pains in rectum, and beating also, and as the wall of the vagina posteriorly is the wall of the rectum anteriorly it seems to me that the diagnosis would have been more scientific and correct, if the vagina had not been mentioned, as it had probably little or nothing to do with the case. Many things in one's daily food, a little nutmeg, for instance, may temporarily remove an attack of piles, or they may pass off, as this attack did for a time, by an increased action of the liver producing bilious diarrhœa, only to come on again if the case were not cured. Next we have given us a long account of knife pains in rectum, or shooting, *but in the rectum now* (evidently the *vagina* pains before mentioned); despondent (no wonder), numbness of legs, feels better walking, hands and feet cold, pain after stool, mental incoherence, thoughts not ready, all of which again strongly point to Sulphur, and yet this poor lady is kept suffering more or less from the 16th of October to 27th of December, when, we are told, the *climax* of pain came, and the *physician* saw he had committed *a fatal error* in giving Kali-carb. and then makes another by giving Hamamelis$^{cm}$. What Chloroform has to do with such a case is difficult to see. It almost seems that he would rather have let the poor lady suffer than give anything short of a hundred-thousandth potency. At last we come to the 19th of January, 1884, more than *three months'* treatment and absolutely nothing done; now the patient develops a "key-note" of Silicea—*stool feels as if it slipped back* (the probability whenever this sensation occurs is that a fold of the rectum is protruded by straining and slips back) consequently on apparently that symptom alone Silicea$^{cm}$ is given. Now mark, the first dose was given at 5 P. M. and at 6 P. M. an enormous stool was voided, which he takes the credit of having caused to be passed in one hour with Silicea$^{cm}$, irrespective of the fact as stated that before the Silicea was given the patient had the *greatest difficulty in preventing a motion*, some stool having passed *involuntarily*. Comment is needless, but even supposing the Silicea did this, it has taken him *three months to find the remedy*. Surely, Mr. Editor, you will give us something better than this next time.

Now, to take an intelligent view of this case, I think few would disagree with me in saying that the piles were owing to pelvic congestion, caused by the pregnant state, the effects of which had not quite subsided, a condition of things that often produce piles by mechanical pressure. The child, we are told, was born in September (*I wonder whether it was a thirteen months' baby*) and the piles gradually went away of themselves, which they would probably do in any similar case in three months, often in less time, *the treatment they received not affecting them in the least*. Piles of the most acute and painful kind are *generally* cured in two or three weeks when the *true* homœopathic remedy is given, which in this case seemed to be Sulphur. I have seldom had an acute case that did not get well in from two to four weeks. So much for that *physician* who seems to think he can teach better and wiser men than himself. As one of the faithful, allow me, Mr. Editor, to sign myself

BUFFALO.

